# Extra-epitopic hepatitis C virus polymorphisms confer resistance to broadly neutralizing antibodies by modulating binding to scavenger receptor B1

**DOI:** 10.1371/journal.ppat.1006235

**Published:** 2017-02-24

**Authors:** Ramy El-Diwany, Valerie J. Cohen, Madeleine C. Mankowski, Lisa N. Wasilewski, Jillian K. Brady, Anna E. Snider, William O. Osburn, Ben Murrell, Stuart C. Ray, Justin R. Bailey

**Affiliations:** 1 Department of Medicine, Johns Hopkins University School of Medicine, Baltimore, Maryland, United States of America; 2 Department of Medicine, University of California, San Diego, La Jolla, California, United States of America; 3 Department of Oncology, Johns Hopkins University School of Medicine, Baltimore, Maryland, United States of America; The University of Chicago, UNITED STATES

## Abstract

Broadly-neutralizing monoclonal antibodies (bNAbs) may guide vaccine development for highly variable viruses including hepatitis C virus (HCV), since they target conserved viral epitopes that could serve as vaccine antigens. However, HCV resistance to bNAbs could reduce the efficacy of a vaccine. HC33.4 and AR4A are two of the most potent anti-HCV human bNAbs characterized to date, binding to highly conserved epitopes near the amino- and carboxy-terminus of HCV envelope (E2) protein, respectively. Given their distinct epitopes, it was surprising that these bNAbs showed similar neutralization profiles across a panel of natural HCV isolates, suggesting that some viral polymorphisms may confer resistance to both bNAbs. To investigate this resistance, we developed a large, diverse panel of natural HCV envelope variants and a novel computational method to identify bNAb resistance polymorphisms in envelope proteins (E1 and E2). By measuring neutralization of a panel of HCV pseudoparticles by 10 μg/mL of each bNAb, we identified E1E2 variants with resistance to one or both bNAbs, despite 100% conservation of the AR4A binding epitope across the panel. We discovered polymorphisms outside of either binding epitope that modulate resistance to both bNAbs by altering E2 binding to the HCV co-receptor, scavenger receptor B1 (SR-B1). This study is focused on a mode of neutralization escape not addressed by conventional analysis of epitope conservation, highlighting the contribution of extra-epitopic polymorphisms to bNAb resistance and presenting a novel mechanism by which HCV might persist even in the face of an antibody response targeting multiple conserved epitopes.

## Author summary

Generation of an immune response that is protective against a wide variety of naturally occurring isolates is necessary for vaccines against highly variable viruses like hepatitis C virus (HCV). Two broadly neutralizing human monoclonal antibodies, HC33.4 and AR4A, neutralize multiple highly divergent HCV isolates, raising hope that a vaccine against HCV is possible. Previous reports have defined the distinct, highly conserved sites on the viral envelope proteins where these antibodies bind. However, little is known about naturally occurring variation in sensitivity of different HCV isolates to these antibodies. We developed a high throughput assay and computational algorithm to evaluate over 100 naturally occurring HCV variants for their sensitivity to these two antibodies, identifying several resistance polymorphisms to each antibody which do not fall within their mapped binding sites. Furthermore, two of these polymorphisms modulate resistance to both antibodies by enhancing or reducing envelope protein binding to HCV co-receptor scavenger receptor B1 (SR-B1). By developing this broadly applicable platform, we have shown the important neutralization resistance conferred by changes distant from antibody binding sites, presenting a potential mechanism by which HCV might persist even in the face of an antibody response targeting multiple conserved sites.

## Introduction

Hepatitis C virus (HCV) infects over 170 million people worldwide [1] and kills more people in the United States annually than HIV [2]. Appalachian regions of the United States saw a >350% increase in the number of new HCV infections from 2009–2012 [3] and recent outbreaks in the United States have been attributed to the rapid increase in injection drug use [4]. While direct-acting antiviral (DAA) therapy has revolutionized care for patients with HCV, control of the HCV pandemic remains challenging due to frequent reinfection in high-risk individuals who have achieved a sustained virologic response after DAA therapy [5], transmission of NS5A inhibitor-resistant HCV variants from individuals failing DAA therapy [6], and the high proportion (~50%) of infected individuals who are unaware asymptomatic carriers [7].

A major goal for the development of a prophylactic vaccine against HCV is stimulation of an immune response that is protective against a wide range of naturally occurring viral variants [8,9], which is a daunting challenge given the enormous genetic diversity of HCV [10–18]. Broadly neutralizing antibodies (bNAbs) are a useful guide for vaccine development, since they bind to relatively conserved viral epitopes, prevent successful entry of diverse HCV isolates, and have been associated with spontaneous clearance of HCV [19]. Despite the relative conservation of bNAb epitopes, polymorphisms conferring resistance to various bNAbs have been identified [20–24], and increasing evidence has shown that polymorphisms distant from bNAb binding sites can modulate E1E2 resistance [20,22,24]. BNAb resistance polymorphisms have been identified by various methods, including alanine-scanning mutagenesis, mapping of longitudinal sequence evolution in infected humans [22], and passage of replication competent virus (HCVcc) in vitro in the presence of bNAbs [21,23], but an efficient method to identify common naturally-occurring resistance polymorphisms in circulating E1E2 variants has not been available.

Recently, we and others have observed significant variation in sensitivity of natural E1E2 variants to a diverse panel of monoclonal bNAbs and HCV-infected sera [24,25]. When we compared the rank orders of neutralization of a diverse array of 19 genotype 1 HCVpp by individual bNAbs, distinct relationships between antibodies were observed, allowing grouping of all bNAbs into three distinct clusters of functionally-related antibodies, and suggesting that common E1E2 determinants of neutralization sensitivity are shared between bNAbs within each cluster [24]. In that study, we identified E2 polymorphisms conferring resistance to most antibodies falling in a group we called neutralization cluster 1, which included bNAbs that are known to target the CD81-binding site of E2.

We were previously unable to explain the functional relationship between antibodies in a second group that we called neutralization cluster 2. Surprisingly, this cluster includes the potent human bNAbs HC33.4 and AR4A, although their described binding epitopes are at opposite termini of the E2 protein [26,27]. HC33.4 is a human monoclonal antibody that binds to a continuous epitope near the N-terminus of E2, at amino acids (aa) 412–423, commonly known as ‘epitope I’ [28]. Recently, aa 408 was also shown to be a HC33.4 binding residue [29]. AR4A, also a human monoclonal antibody, binds to a conformational epitope including the C-terminal, membrane proximal region of E2 as well as residues on E1. BNAbs like HC33.4 targeting ‘epitope I’ have been shown to neutralize HCV by blocking E2 interaction with CD81 [30–35], while AR4A does not appear to interfere with this interaction [26].

Despite the clearly distinct binding epitopes of these two bNAbs, and possibly differing mechanisms of neutralization, we hypothesized that shared E1E2 resistance polymorphisms to these antibodies would explain the unexpected correlation between neutralization profiles of HC33.4 and AR4A. No obvious polymorphisms mediating this effect were identified in a small panel of E1E2 variants with varying HC33.4 and AR4A sensitivities, so we developed a larger panel of more than 100 E1E2 variants as well as a statistical approach to identify natural polymorphisms that were associated with resistance to each bNAb. Using these tools, we identified polymorphisms conferring resistance to HC33.4 and AR4A individually, as well as polymorphisms outside of either binding epitope that confer resistance to both bNAbs by modulating binding to the HCV co-receptor, scavenger receptor B1 (SR-B1).

## Results

### The library of envelopes contains 113 distinct, functional, naturally occurring E1E2 clones

**Fig 1:**
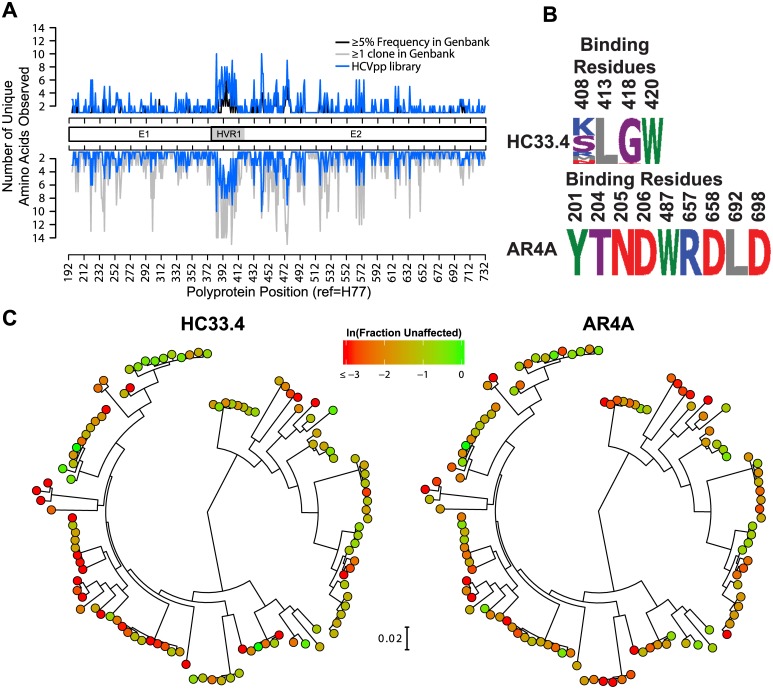
Construction of an HCV E1E2 panel for neutralizing antibody breadth testing and sequence prediction of neutralizing antibody resistance polymorphisms. (**A**) Number of different amino acids present at each position in the 113 variant E1E2 panel (blue line). Regions spanning E1, hypervariable region 1 (HVR1), and E2 are indicated. For comparison, black line in the upper plot shows the number amino acids represented at each position by at least 5% of the sequences in a reference panel of 643 HCV genotype 1 isolates from GenBank. Gray line in the lower plot shows the number amino acids represented at each position by at least one sequence in the reference panel of 634 HCV genotype 1 isolates from GenBank. (**B**) Variation in the E1E2 panel of known critical binding residues for HC33.4 and AR4A. Numbers indicate the polyprotein position of each amino acid. Height of each amino acid is proportional to its frequency in the panel. Letters are standard IUPAC amino acid abbreviations. (**C**) Phylogenetic tree of E1E2 amino acid sequences of the 113 variant panel, determined by maximum likelihood, shown with the distances drawn to scale. Clones are colored according to their sensitivity to neutralization: ln(Fraction Unaffected (F_u_) by 10 μg/mL of HC33.4 (left) and AR4A (right)). F_u_ is infection in the presence of 10 μg/mL of bNAb/infection in the presence of nonspecific human IgG.

To construct a library of E1E2 genes to predict relationships between amino acid sequence and neutralization sensitivity, we cloned more than 700 naturally occurring HCV genotype 1 E1E2 genes. Of these cloned E1E2s, 113 produced HCV pseudoparticles (HCVpp) that were functional in repeated tests when co-transfected with an HIV NL4.3Δenv-Luc reporter genome, as previously described [24]. The resulting library includes 71 subtype 1a and 42 subtype 1b E1E2 variants isolated from a total of 27 unique donors. It is not known why the majority of cloned E1E2 variants were nonfunctional in HCVpp, but this has also been observed in other studies [36]. We analyzed genetic variation in our functional E1E2 panel to confirm that it is representative of circulating strains. Loci of greatest amino acid variation of the panel across E1E2 mirror those of a reference panel of 643 genotype 1 HCV isolates from GenBank, with the greatest amino acid diversity observed in hypervariable region 1 (HVR1) of E2 (Fig 1A). As many as 9 possible amino acids are represented at some E1E2 loci. Overall, this neutralization panel of functional E1E2 variants contains 97% of amino acid polymorphisms present at ≥5% frequency in the Genbank genotype 1 reference panel.

### The panel of HCVpp displays large variation in sensitivity to neutralization by HC33.4 and AR4A despite conservation of binding epitopes

We first assessed variation at the known binding epitopes of the two bNAbs across the HCVpp panel. The HC33.4 epitope varied at position 408, while the AR4A epitope was 100% conserved across the panel at all known binding residues (Fig 1B). We then quantitated the fraction unaffected (F_u_) of each HCVpp in the presence of each bNAb by measuring hepatoma cell entry of HCVpp in the presence of 10 μg/mL HC33.4 or AR4A relative to entry in the presence of nonspecific human IgG (Fig 1C). Neutralization was assessed at a single concentration of bNAb rather than with serial bNAb dilutions in order to increase the throughput of the assay and to minimize the quantity of bNAb required. We have previously shown that F_u_ values measured by this method are reliably quantitative, as they correlate significantly with IC_50_ values calculated from neutralization curves of the same HCVpp/bNAb combinations as well as with IC_50_ values calculated from neutralization curves of replication competent virus (HCVcc) [24]. Another recent study also confirmed strong concordance between neutralization results obtained using HCVpp or HCVcc [36]. Each bNAb showed a more than 100-fold variation in neutralization across the HCVpp panel, which was surprising given the conservation of the bNAb binding epitopes. HC33.4 and AR4A were associated with a median (min-max) F_u_ of 0.22 (0.003–1.1) and 0.17 (0.01–1.15), respectively. Using a cutoff of F_u_<0.5, which is roughly equivalent to an IC_50_ cutoff of 10 μg/mL, HC33.4 and AR4A neutralized 88% and 85.8% of the HCVpp panel, respectively.

### A positive correlation was observed between neutralization profiles of AR4A and HC33.4

**Fig 2:**
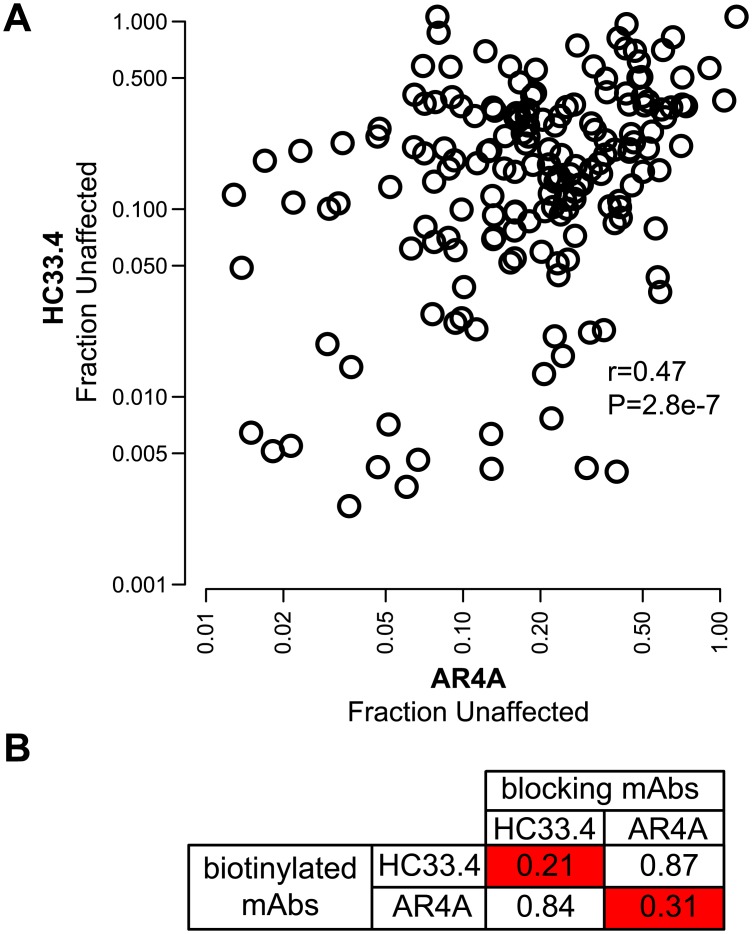
Relative resistance of diverse HCVpp to neutralization by HC33.4 and AR4A shows a significant positive correlation, which is not the result of shared binding sites. (**A**) Each point indicates mean fraction unaffected (F_u_) of a single HCvpp by 10 μg/mL of HC33.4 on the x-axis and AR4A on the y-axis, measured in duplicate. F_u_ is infection in the presence of 10 μg/mL of bNAb/infection in the presence of nonspecific human IgG. R- and p-values determined by Spearman correlation. (**B**) HC33.4 and AR4A do not compete for binding to E1E2. Values represent binding of biotinylated bNAbs to 1a154 (H77) E1E2 in an ELISA in the presence of the indicated blocking antibody relative to binding in the absence of blocking antibody. Combinations showing >50% reduction in binding of the biotinylated bNAb are marked in red.

As we previously observed using a panel of 19 HCVpp [24], there was a significant positive correlation between the rank order of sensitivity of the 113 HCVpp in this panel to HC33.4 and AR4A (r = 0.44 p = 7e-7) (Fig 2A). We identified E1E2 variants with exquisite sensitivity to both bNAbs (F_u_ < 0.02 with either bNAb) as well as variants with high level resistance to both bNAbs (F_u_ >1), with other E1E2 variants distributed between those extremes. This correlation between neutralization profiles of the two bNAbs was surprising, given that they do not share binding residues (Fig 1B).

**Fig 3:**
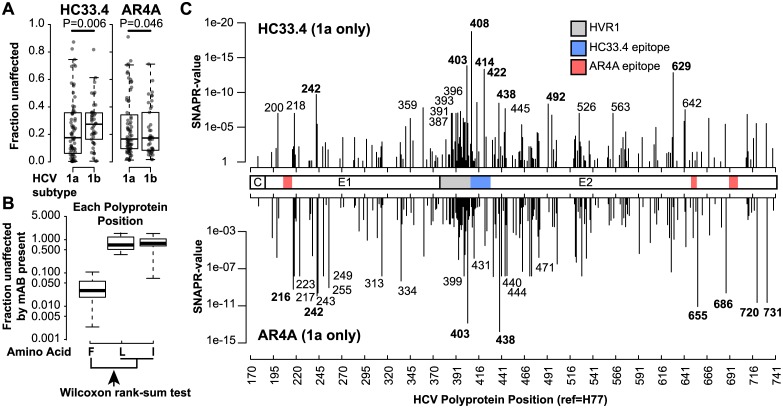
Subject-adjusted Neutralizing Antibody Prediction of Resistance-polymorphisms (SNAPR): Prediction of polymorphisms mediating neutralization resistance. (**A**) Differences in HC33.4 and AR4A neutralization of HCVpp by subtype. Each data point indicates the mean F_u_ of an individual HCVpp, measured in duplicate. Boxes are interquartile range, and horizontal lines are medians. P-values calculated by Wilcoxon rank sum test. (**B**) Illustration of the SNAPR method: At each E1E2 alignment position, isolates were divided into groups according to amino acid present. Fraction unaffected (F_u_, infection in the presence of bNAb/infection in the presence of nonspecific IgG) values of HCVpp from the most sensitive group (as determine by the medians) was compared to the F_u_ of HCVpp with any other amino acid at that position by Wilcoxon rank-sum test to generate SNAPR-values. (**C**) SNAPR-values across E1E2 determined using only subtype 1a HCVpp neutralized by HC33.4 or AR4A. The 8 positions with lowest SNAPR-values for HC33.4 and AR4A are in bold print. Previously defined HC33.4 and AR4A binding epitopes are indicated (blue and pink), as is hypervariable region 1 (HVR1) (gray).

To further confirm that the two bNAbs bind to distinct epitopes, we performed binding competition assays between the two bNAbs. E1E2 protein-coated ELISA wells were pre-incubated with a high concentration of either HC33.4 or AR4A (blocking bNAbs), followed by biotinylated HC33.4 or AR4A at a concentration selected to give 50% of maximal binding (EC_50_), with binding of the biotinylated bNAb detected using streptavidin-horseradish peroxidase. A ratio of binding of each biotinylated bNAb in the presence of blocking bNAb divided by binding in the absence of blocking bNAb was calculated (Fig 2B). As expected, each bNAb competed for binding with itself, but HC33.4 and AR4A showed minimal competition for E1E2 binding with each other, confirming that the bNAbs bind to distinct epitopes. In spite of their shared resistance pattern, HC33.4 more potently neutralized subtype 1a than subtype 1b HCVpp (F_u_ median = 0.17 for 1a vs. 0.27 for 1b, p = 0.002), while AR4A displayed minimal difference in subtype neutralization (Fig 3A). The significant positive correlation between HC33.4 and AR4A neutralization profiles observed with the full HCVpp panel was also observed on analysis of the subtype 1a-only (r = 0.39, p = 7e-4) and subtype 1b-only (r = 0.69, p = 1e-6) subsets of the panel (S1 Fig)

### Subject-adjusted Neutralizing Antibody Prediction of Resistance-polymorphisms (SNAPR) and the Least Absolute Shrinkage and Selection Operator (LASSO) models: Using variation in sequence and neutralization sensitivity to predict resistance polymorphisms in the HCV envelope

We used novel (SNAPR) and established (LASSO) methods to identify E1E2 sequence determinants of resistance to HC33.4 and AR4A. An E1E2 amino acid alignment was generated including sequences of each of the 113 variants in the panel. For the SNAPR method, due to the higher degree of similarity among E1E2 variants originating from the same HCV-infected donor, and a variable number of E1E2 variants contributed by each donor, neutralization and sequence data from variants from underrepresented donors were randomly selected for replication until the number of data points in the analysis representing each individual donor was identical. For each amino acid position across E1E2, all HCVpp were divided into groups according to the amino acid occupying that position, and the amino acid associated with lowest median F_u_ (greatest neutralization sensitivity) for each bNAb was identified. For each bNAb, at each position, a nonparametric (Wilcoxon rank-sum) test was used to compare the neutralization values of all HCVpp with E1E2 carrying the amino acid associated with the lowest mean F_u_ with the neutralization values of all of the other variants, generating a “SNAPR value” for each position (Fig 3B). While the replication of some sequence and neutralization data reduces the effect of over-representation of some donors, the method also artificially inflates the sample size with data that are not independent, so calculated p-values are artificially low. Therefore, the p-values themselves cannot be used to determine whether variation in neutralization sensitivity at an individual position is statistically significant. Rather, these "SNAPR-values" provide a metric for comparison between effects at different polyprotein positions. Because of the potential for subtype differences to dominate findings, grouped genotype (S2 Fig) and subtype 1a only analyses were performed separately for further focused investigation. SNAPR-values spanned approximately 10 and 20 orders of magnitude for HC33.4 and AR4A, respectively (Fig 3C).

We also re-analyzed the neutralization and sequence data using a method that considers multiple residues in combination—LASSO, without the replication of data required for the SNAPR analysis [37]. Of the 20 most likely resistance polymorphism position predictions from SNAPR for HC33.4 and AR4A (Table 1) 5 positions (for HC33.4) and 5 positions (for AR4A) were also among the 20 and 18 most likely LASSO predictions, respectively. Of note, position 408 was predicted by SNAPR to modulate resistance to HC33.4 but not AR4A, which supports sensitivity and specificity of the SNAPR analysis since lysine (K) 408 is a known binding residue for HC33.4 but not AR4A, and it is the only known binding residue for either bNAb that varies significantly across the neutralization panel. Position 408 was not among the top 20 LASSO predictions for HC33.4.

**Table 1:** Agreement between Subject-adjusted (SNAPR) and phylogenetic-adjusted (LASSO) resistance polymorphism predictions made using the same HCVpp neutralization data set.

HC33.4			AR4A		
polymorphism polyprotein position^1^	SNAPR value	LASSO Rank^2^	polymorphism polyprotein position^1^	SNAPR value	LASSO Rank^2^
**408**	**1.82E-19**	**>20**	**438**	**1.68E-14**	**>18**
**403**	**1.48E-14**	**1**	**403**	**1.40E-13**	**1**
**422**	**4.85E-14**	**>20**	**655**	**8.34E-12**	**12**
**629**	**1.47E-13**	**>20**	**720**	**2.32E-11**	**>18**
**242**	**2.00E-10**	**13**	**731**	**2.32E-11**	**>18**
**414**	**2.82E-09**	**>20**	**242**	**1.20E-10**	**8**
**438**	**3.55E-09**	**>20**	**686**	**2.47E-10**	**>18**
**492**	**4.74E-09**	**>20**	**216**	**6.17E-10**	**>18**
359	1.62E-08	18	243	7.47E-10	>18
445	2.17E-08	>20	255	9.04E-10	>18
642	3.07E-08	>20	334	4.80E-09	10
396	5.45E-08	4	431	1.41E-08	7
393	9.21E-08	2	217	1.65E-08	>18
200	1.03E-07	>20	223	1.65E-08	>18
218	1.03E-07	>20	249	1.65E-08	>18
387	1.03E-07	>20	313	1.65E-08	>18
391	1.03E-07	>20	399	1.65E-08	>18
526	1.03E-07	>20	440	1.65E-08	>18
563	1.03E-07	>20	444	1.65E-08	>18
710	1.49E-07	>20	471	1.65E-08	>18

^**1**^The 20 most likely resistance polymorphism predictions by SNAPR ranked from lowest to highest SNAPR value. Predictions selected for confirmation by site-directed mutagenesis are in bold.

^**2**^ Rank of each position based on LASSO coefficient. Sites with no effect on the LASSO model (i.e. Inferred coefficients = 0) are shown as >20 (HC33.4) or >18 (AR4A).

### Polymorphisms at polyprotein positions 242, 403 and 438 are predicted determinants of neutralization sensitivity for both HC33.4 and AR4A

Given the distinct binding epitopes of HC33.4 and AR4A (Figs 1B and 3C), and the imperfect correlation between neutralization profiles of the bNAbs (Fig 2A), it is not surprising that many of the loci predicted to modulate sensitivity to HC33.4 and AR4A are not shared. Interestingly, SNAPR predicted positions 242, 403, and 438 as determinants of neutralization sensitivity for both HC33.4 and AR4A. Positions 242 and 403 were also predicted by LASSO to be determinants of sensitivity for both bNAbs (Table 1).

**Fig 4:**
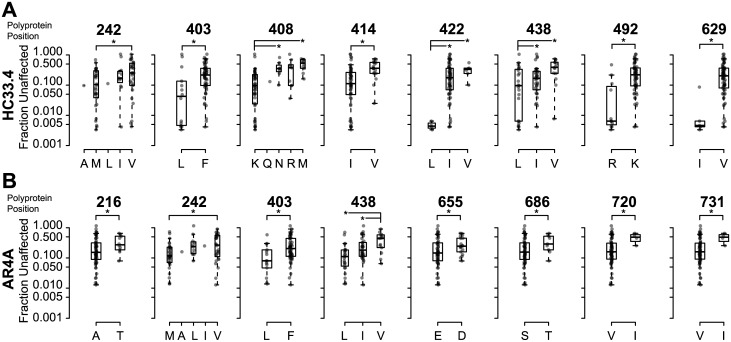
Comparison of F_u_ of HCVpp segregated by the amino acid present at positions predicted by SNAPR to influence bNAb resistance. Boxplots showing the F_u_ values for all isolates grouped by amino acid present at the indicated position in the presence of HC33.4 (**A**) and AR4A (**B**) in order of their polyprotein position. Each data point indicates the mean F_u_ of an individual HCVpp, measured in duplicate. Boxes are interquartile range, and horizontal lines are medians. The eight positions with lowest SNAPR-values for each bNAb are shown. The letters shown are standard IUPAC amino acid abbreviations. F_u_ of HCVpp with the indicated polymorphisms were compared by Wilcoxon rank-sum test. (*, p<0.05).

To further investigate the 8 most likely SNAPR predictions for HC33.4 and AR4A, we compared F_u_ values of HCVpp grouped by the amino acid present at each position, without the replicated neutralization data included in the SNAPR analysis (Fig 4). In the analysis for HC33.4, E1E2 variants with methionine (M) vs. valine (V) at position 242 showed significant differences in neutralization sensitivity (median F_u_ 0.10 vs. 0.25, p = 0.02) (Fig 4A). For AR4A, M vs. V at position 242 were also associated with significant differences in neutralization sensitivity (median F_u_ 0.12 vs. 0.27, p = 0.02) (Fig 4B). Variation at the 242 position resulted in the 13^th^ and 8^th^ most extreme LASSO coefficients of any position in E1E2 for HC33.4 and AR4A, respectively (Table 1).

Variants with leucine (L) vs. phenylalanine (F) at position 403 showed significant differences in neutralization sensitivity to HC33.4 (median F_u_ 0.042 vs. 0.22, p = 1E-3). (Fig 4A). For AR4A, L vs. F at position 403 were also associated with significant differences in neutralization sensitivity (median F_u_ 0.08 vs. 0.20, p = 0.01) (Fig 4B). Variation at the 403 position resulted in the most extreme LASSO coefficients of any position in E1E2 for HC33.4 and AR4A (Table 1).

For HC33.4, E1E2 variants with leucine (L) vs. valine (V) at position 438 also showed significant differences in neutralization sensitivity (median F_u_ 0.096 vs. 0.39, p = 3E-3). (Fig 4A). For AR4A, L vs. V at position 438 were also associated with significant differences in neutralization sensitivity (median F_u_ 0.11 vs. 0.44, p = 0.01) (Fig 4B).

### Reciprocal site-directed mutations of neutralization-sensitive and -resistant E1E2 variants validate SNAPR-predicted resistance polymorphisms

**Fig 5:**
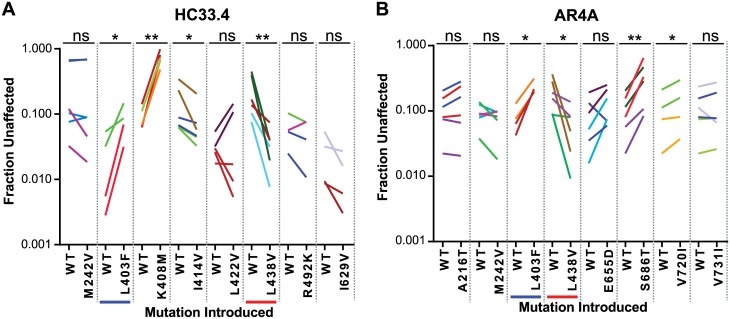
Site-directed mutagenesis confirms multiple SNAPR-predicted resistance polymorphisms. Putative resistance polymorphisms were introduced by site-directed mutagenesis into multiple distinct wild type (WT) E1E2 variants in which they were not naturally present. These WT variants were each from subtype 1a and differed from each other prior to mutagenesis by an average of 42 amino acids (7%). WT and mutated E1E2 variants were used to produce HCVpp, which were tested for neutralization by 10 μg/mL of HC33.4 (**A**) or AR4A (**B**). Fraction unaffected is the infection in the presence of 10 μg/mL of neutralizing antibody/infection in the presence of nonspecific IgG. Each mutation was introduced into 2–3 distinct E1E2 variants and neutralization effect of each mutation was tested in at least 4 independent experiments performed in duplicate. Each line indicates an independent experiment comparing a WT HCVpp to the corresponding mutant version, and different colors indicate different E1E2 variants. WT HCVpp and corresponding mutant HCVpp neutralization were compared by paired, two-sided T test. (ns, not significant; *, p<0.05; **, p<0.005).

To test SNAPR predictions, putative resistance polymorphisms at the 8 positions with the lowest SNAPR-values for each bNAb were introduced individually by site directed mutagenesis into 2–3 distinct wild type (WT) E1E2 variants in which they were not naturally present. These WT variants were each from subtype 1a and differed from each other prior to mutagenesis by an average of 42 amino acids (7%). Mutated E1E2 and corresponding WT variants were used to produce HCVpp, which were tested for neutralization by HC33.4 (Fig 5A) or AR4A (Fig 5B). To control for experimental variation between HCVpp neutralization experiments, neutralization of each mutated and WT HCVpp pair was tested in at least 2 independent experiments. Experiments were considered independent only if independently produced HCVpp preparations (transfections) were used and independent neutralization assays were performed. M242V, L403F and L438V were predicted to modulate resistance to both HC33.4 and AR4A, so these mutations were tested for effect on each bNAb. Four of the 8 polymorphisms predicted by SNAPR to confer resistance to HC33.4 showed statistically significant effects after introduction by site directed mutagenesis. Notably, mutation of lysine (K) 408 to methionine (M) led to an increase in resistance (WT mean F_u_ of 0.09 vs. K408M mean F_u_ of 0.72, p<0.0001), which was expected since K408 was recently identified by alanine scanning as a binding residue for HC33.4 [29]. Mutation of leucine (L) 403 to phenylalanine (F) also led to a significant increase in resistance to HC33.4. Curiously, mutation of L438 to valine (V), which was predicted by SNAPR to confer resistance to HC33.4, instead conferred significantly increased sensitivity to the bNAb (WT mean F_u_ 0.22 vs. L438V mean F_u_ 0.04, p = 0.004).

Four of the 8 polymorphisms predicted to confer resistance to AR4A also showed statistically significant effects after introduction by site directed mutagenesis. As with HC33.4, L403F conferred significant resistance to AR4A neutralization (WT mean F_u_ 0.08 vs. L403F mean F_u_ 0.23, p = 0.007), and L438V conferred increased AR4A sensitivity (WT mean F_u_ 0.20 vs. L438V mean F_u_ 0.06, p = 0.03). Notably, mutation of serine (S) 686 to threonine (T) and valine (V) 720 to isoleucine (I) also conferred significant resistance to AR4A. Neither S686 nor V720 fall at a known binding residue for AR4A, but they are 6 and 22 amino acids from known AR4A binding residues, respectively. Though polymorphisms at 242 were also predicted by SNAPR and LASSO to be determinants of resistance for AR4A and HC33.4 (Fig 3C; Table 1), mutagenesis at this position did not confer a significant change in sensitivity to either antibody. Taken together, these results confirm that L403F and L438V modulate sensitivity to neutralization by both HC33.4 and AR4A.

**Fig 6:**
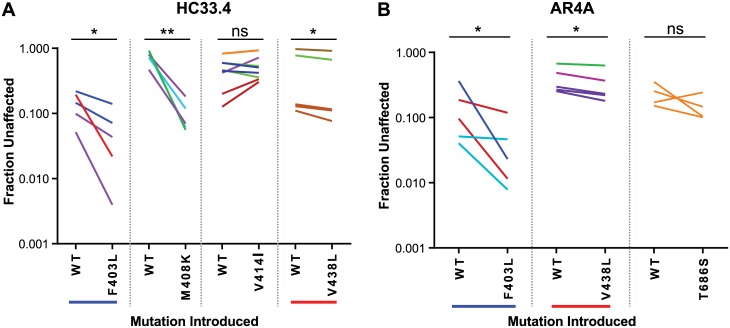
Reversion of naturally-existing resistance polymorphisms confirms their phenotype. Putative resistance polymorphisms that were already validated by introduction into neutralization sensitive E1E2 variants were reverted in other E1E2 variants where they were already naturally present. Wild type (WT) and mutated E1E2 variants were used to produce HCVpp, which were tested for neutralization by 10 μg/mL of HC33.4 (**A**) or AR4A (**B**). Fraction unaffected is the infection in the presence of 10 μg/mL of neutralizing antibody/infection in the presence of nonspecific IgG. Each mutation was introduced into 2–5 distinct E1E2 variants, except T686S, which could be introduced into only one E1E2 variant, and neutralization effect of each mutation was tested in at least 4 independent experiments performed in duplicate. Each line indicates an independent experiment comparing a WT HCVpp to the corresponding mutant version, and different colors indicate different E1E2 variants. Wild type (WT) and corresponding mutant HCVpp neutralization were compared by paired, two-sided T test. (ns, not significant; *, p<0.05; **, p<0.005).

All resistance polymorphisms except V720I that had been validated by introduction into neutralization sensitive E1E2 variants were reverted in 2–5 distinct E1E2 variants where they were naturally present (Fig 6). Mutated E1E2 variants and corresponding WT variants were used to produce HCVpp, which were tested for neutralization by HC33.4 (Fig 6A) or AR4A (Fig 6B). Three of four polymorphisms that showed an effect on HC33.4 when introduced into neutralization sensitive E1E2 variants also showed a significant effect when reverted in E1E2 variants where they were already naturally present. Mutation of M408 to the known HC33.4 binding residue, K, resulted in significantly increased sensitivity to HC33.4 neutralization (Wild type mean F_u_ 0.75 vs. M408K F_u_ 0.10, p = 0.001). Mutation of F403 to L also increased sensitivity to HC33.4. Mutation of V438 to L also conferred a small but significant increase in HC33.4 sensitivity (Wild type mean F_u_ 0.43 vs. V438L F_u_ 0.38, p = 0.02). This was unexpected because in other E1E2 variants, the reciprocal mutation of L438 to V had also conferred increased neutralization sensitivity, but the magnitude of the effect of V438L was very small (mean F_u_ fold change of 0.9) relative to the magnitude of the effect of L438V (mean F_u_ fold change of 0.2)

Two of three polymorphisms that showed an effect on AR4A when introduced into neutralization sensitive E1E2 variants also showed a significant effect when reverted in E1E2 variants where they were already naturally present. Most notably, mutation of F403 to L and mutation of V438 to L conferred increased sensitivity to AR4A, just as they had for HC33.4. Taken together, these results show that mutation of L403 to F and mutation of F403 to L confer reciprocal neutralization resistance and sensitivity effects on both HC33.4 and AR4A, while mutation of L438 to V in some E1E2 variants and V438 to L in others confers increased sensitivity to neutralization by both HC33.4 and AR4A.

### The magnitude of change in neutralization sensitivity of HCVpp and HCVcc conferred by L403F and L438V is similar for HC33.4 and AR4A

**Fig 7:**
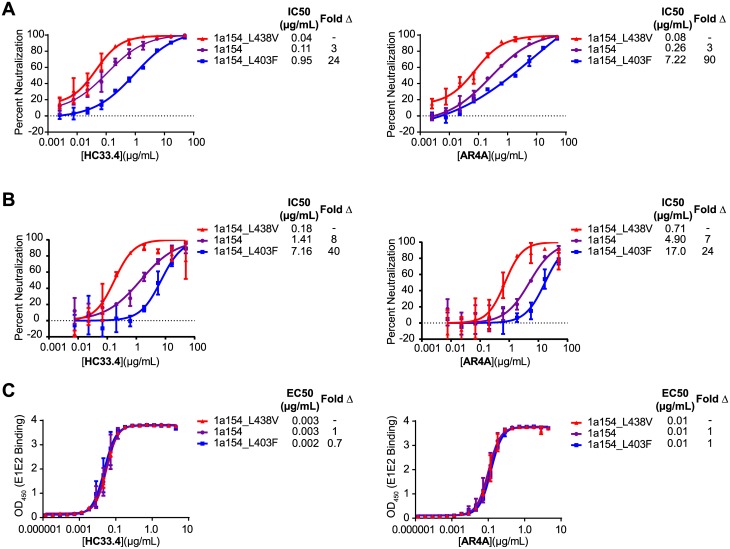
L403F and L438V confer similar-magnitude changes in HCVpp and HCVcc neutralization sensitivity to HC33.4 and AR4A, but no changes in bNAb binding. (**A**) L403F confers neutralization resistance and L438V confers increased neutralization sensitivity to both HC33.4 and AR4A. The indicated mutations were introduced into E1E2 variant 1a154 (H77), and HCVpp were generated. Neutralization of wild type and mutant HCVpp by serial dilutions of HC33.4 or AR4A was measured. Each point is the mean of two replicate values, and error bars indicate standard deviations. 50% inhibitory concentration (IC_50_) of each bNAb/HCVpp combination and fold change in IC_50_ relative to 1a154_L438V are indicated. **(B**) Neutralization of replication competent cell culture virus (HCVcc) generated with wild type 1a154 (H77) or mutant E1E2. Each point is the mean of triplicate values, and error bars indicate standard deviations. 50% inhibitory concentration (IC_50_) of each bNAb/HCVcc combination and fold change in IC_50_ relative to 1a154_L438V are indicated. (**C**) L403F and L438V do not change binding of HC33.4 or AR4A to E1E2 protein. Binding to wild type and mutant E1E2 protein by serial dilutions of HC33.4 or AR4A was measured in an ELISA. Each point is the mean binding measured in two independent experiments performed in duplicate, with error bars indicating standard deviations between experiments, except binding at the lowest 7 bNAb concentrations, which was tested in only one experiment. BNAb concentration resulting in half-maximal binding (EC_50_) of each bNAb/E1E2 combination and fold change in EC_50_ relative to 1a154_L438V are indicated.

To measure the magnitude of the effect of L403F and L438V mutations on neutralization sensitivity, we measured neutralization of wild type 1a154 (H77), 1a154_L438V, and 1a154_L403F HCVpp by serial dilutions of HC33.4 and AR4A (Fig 7A). As expected, the 1a154_L438V HCVpp variant was most sensitive to neutralization. Wild type 1a154 HCVpp showed a 3-fold increase in IC_50_ relative to 1a154_L438V for both antibodies, and 1a154_L403F HCVpp showed a 24-fold increase in IC_50_ relative to 1a154_L438V HCVpp for HC33.4 and a 90-fold increase in IC_50_ for AR4A. We also confirmed the resistance phenotypes of the mutations using replication competent cell culture virus (HCVcc) (Fig 7B). Wild type 1a154, 1a154_L438V, or 1a154_L403F E1E2 genes were cloned into a J6/JFH-1 HCVcc genome lacking E1E2 [38], and replication competent virus was produced from each chimeric strain. HCVcc neutralization results mirrored those observed with HCVpp very closely, with 1a154_L438V most sensitive to neutralization by each bNAb, wild type 1a154 8-fold more resistant to HC33.4 and 7-fold more resistant to AR4A, and 1a154_L403F 40-fold more resistant to HC33.4 and 24-fold more resistant to AR4A.

To understand whether these changes in neutralization sensitivity were mediated by changes in binding of the bNAbs to E1E2, we performed an ELISA to measure binding of serial dilutions of the bNAbs to 1a154_L438V, 1a154, and 1a154_L403F E1E2 proteins (Fig 7C). No significant difference in binding of either bNAb to the E1E2 variants was observed, suggesting that differences in bNAb binding to E1E2 are likely not the mechanism by which L403F and L438V modulate resistance to neutralization by HC33.4 and AR4A.

### L403F and L438V modulate bNAb resistance by altering binding to SR-B1

**Fig 8:**
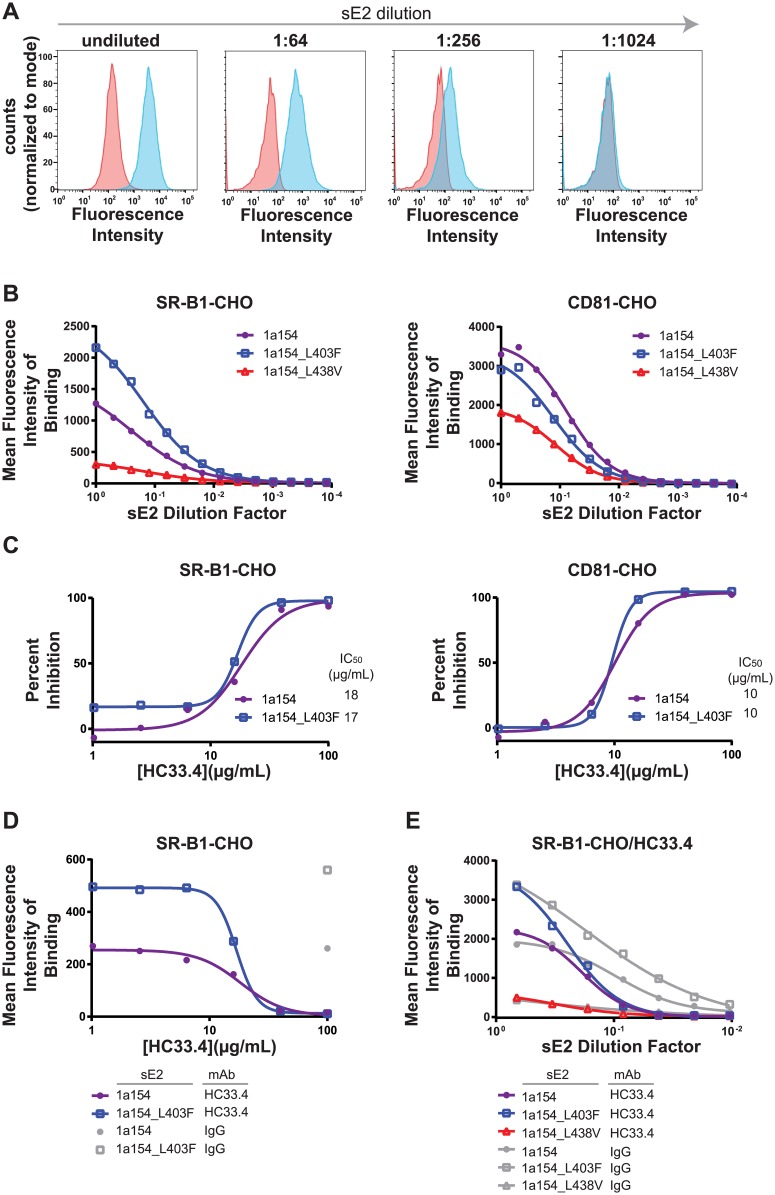
L403F and L438V modulate bNAb resistance by altering binding to SR-B1. **(A)** Binding of serial dilutions of strain 1a154 (H77) soluble E2 (sE2) to CHO cells expressing human CD81 (blue peaks) or to wild type CHO cells that do not express HCV receptors (pink peaks), measured by flow cytometry. (**B**) Binding of serial dilutions of 1a154, 1a154_L403F, or 1a154_L438V sE2 to SR-B1-CHO cells or CD81-CHO cells. Each point was calculated from 10e4 events. Background binding to wild type CHO cells was subtracted from mean fluorescence intensity (MFI) values. sE2 supernatants were normalized for relative sE2 concentration (shown in S3 Fig) prior to dilution. One experiment that is representative of two independent experiments is shown. The second experiment is shown in S4 Fig. (**C**) Percent inhibition of sE2 binding to SR-B1 or CD81 by HC33.4. MFI of binding of a fixed, equivalent concentration of 1a154 or 1a154_L403F sE2 to SR-B1-CHO or CD81-CHO cells in the presence of serial dilutions of mAb HC33.4 or nonspecific human IgG was used to calculate percent inhibition of binding. Each point was calculated from 10e4 events. Background binding to wild type CHO cells was subtracted from mean fluorescence intensity (MFI) values. IC_50_ was calculated by nonlinear regression. (**D**) Binding of equivalent concentrations of sE2 to SR-B1-CHO cells in the presence of serial dilutions of HC33.4. Each point was calculated from 10e4 events. Background binding to wild type CHO cells was subtracted from mean fluorescence intensity (MFI) values. Binding after incubation of each sE2 with 100 μg/mL of nonspecific IgG is shown for reference. (**E**) Binding of serial dilutions of 1a154, 1a154_L403F, or 1a154_L438V sE2 to SR-B1-CHO cells after preincubation of sE2 with 40 μg/mL of HC33.4. Each point was calculated from 10e4 events. Background binding to wild type CHO cells was subtracted from mean fluorescence intensity (MFI) values. sE2 supernatants were normalized for relative sE2 concentration prior to dilution.

Using Chinese hamster ovary (CHO) cells stably expressing either human CD81 or human SR-B1 [33], we investigated relative binding of wild type 1a154 (H77), 1a154_L403F, and 1a154_L438V E2 proteins to these HCV receptors. We used previously described methods to clone these variants without E1 and with replacement of their transmembrane domain with a histidine tag, allowing their expression as soluble E2 (sE2) [39]. Serial dilutions of these soluble proteins were incubated with CD81-CHO, SR-B1-CHO, or wild type CHO cells, then labeled with anti-HIS and fluorescent secondary antibodies to allow detection of binding of sE2 on the cell surface. We were able to quantitate dose-dependent binding of sE2 to both CD81 and SR-B1 using this technique. Fig 8A shows flow cytometry histogram plots of binding of serial dilutions of 1a154 sE2 to CD81-CHO cells, relative to background binding to wild type CHO cells without CD81 or SR-B1.

After normalizing for total sE2 protein input (S3 Fig), we compared binding of serial dilutions of 1a154, 1a154_L403F, and 1a154_L438V sE2 proteins to SR-B1 and CD81 (Fig 8B). Remarkably, we saw a clear increase in binding of 1a154_L403F to SR-B1 relative to binding of wild type 1a154, and we saw a decrease in SR-B1 binding of 1a154_L438V, matching the hierarchy of neutralization resistance of these E2 variants. In comparing binding of the same variants to CD81, we observed a small decrease in binding of 1a154_L403F relative to 1a154, and a large decrease in binding of 1a154_L438V, confirming that the differences between 1a154 and 1a154_L403F binding to SR-B1 are not likely due to differences in protein input.

We next compared binding of a matched, fixed concentration of 1a154 and 1a154_L403F sE2 to SR-B1 and CD81 in the presence of increasing concentrations of nonspecific IgG or HC33.4 (Fig 8C). The 1a154_L438V sE2 variant did not have high enough baseline binding to allow accurate measurement of percent inhibition by HC33.4, and we were also not able to study AR4A in this manner because it requires both E1 and E2 for binding. HC33.4 reduced binding of 1a154 and 1a154_L403F variants to both SR-B1 and CD81 in a dose-dependent manner. It is not surprising that HC33.4 inhibits both SR-B1 and CD81 binding, since a prior study of HC33.4-like antibodies showed that some could block binding to both receptors [33]. The concentrations of HC33.4 inhibiting 50% of binding to SR-B1 or CD81 (IC_50_ values) of 1a154 and 1a154_L403F sE2 were nearly identical, suggesting that the differing affinities of these proteins for SR-B1 and CD81 did not alter the percent binding inhibition of each by equivalent concentrations of mAb.

Unlike HCVpp in neutralization assays, the sE2 variants are normalized for protein concentration, so it is also informative to consider absolute sE2 binding in the presence of mAb. To determine whether modulation of SR-B1 binding could mediate mAb neutralization resistance, we analyzed absolute SR-B1 binding of a fixed concentration of sE2 in the presence of varying concentrations of HC33.4 (Fig 8D), and binding of varying concentrations of sE2 in the presence of a fixed concentration of HC33.4 (Fig 8E). Comparison of sE2 binding of 1a154 and 1a154_L403F in the presence of increasing concentrations of HC33.4 (Fig 8D), showed that 1a154_L403F sE2 bound more SR-B1 than an equivalent concentration of 1a154 sE2 at inhibitory but non-saturating concentrations of HC33.4. We also measured binding of serial dilutions of 1a154, 1a154_L403F, and 1a154_L438V sE2 proteins to SR-B1 after preincubation with a fixed concentration of HC33.4 (40 μg/mL) (Fig 8E). As observed in the absence of antibody, multiple concentrations of 1a154_L403F sE2 incubated with a high concentration of HC33.4 showed greater binding to SR-B1 relative to 1a154 sE2, and 1a154_L438V sE2 showed consistently less binding. Together, these results suggest that, in the presence of inhibitory but non-saturating concentrations of HC33.4, 1a154_L403F sE2 binds more SR-B1 than an equivalent concentration of 1a154 sE2, and 1a154_L438V sE2 binds less, providing a likely mechanism by which these polymorphisms could confer increased resistance or sensitivity, respectively, to mAbs whose mechanism of neutralization is interference with the E2-SR-B1 interaction.

## Discussion

We have developed a high-throughput platform for measurement of neutralizing antibody breadth and prediction of HCV neutralizing antibody resistance polymorphisms. Despite the relative conservation of HC33.4 and AR4A binding epitopes, with 100% conservation of known AR4A binding residues across the panel, we identified E1E2 variants with resistance to one or both bNAbs. We also identified amino acid polymorphisms in E2 conferring resistance to each bNAb individually, as well as polymorphisms outside of both binding epitopes that modulate resistance to both bNAbs. We determined that two of these polymorphisms, L403F and L438V, modulate resistance of both HCVpp and HCVcc to both HC33.4 and AR4A. These mutations increase or reduce E2 binding to SR-B1, identifying a novel mechanism of broad bNAb resistance.

It is interesting that HC33.4 IC_50_ values calculated from inhibition of binding of 1a154 and 1a154_L403F to SR-B1 were nearly equivalent, despite the apparent differences in affinity of the two sE2 variants for SR-B1 (Fig 8C). This could be a limitation in the sensitivity of the binding assay, or alternatively could suggest that HC33.4 binding affinity for sE2 is significantly higher than the affinity of even the 1a154_L403F-SR-B1 interaction. These binding inhibition IC_50_ values were higher than the neutralization IC_50_ values measured for the same variants, likely due to differences in the assays, such as the amount of E2-receptor interaction necessary to generate a detectable signal above background. We show that, despite the equivalent binding inhibition IC_50_ values of 1a154 and 1a154_L403F, differences in sE2 binding to SR-B1 are a likely mechanism of neutralization resistance, since the neutralization resistant variant, 1a154_L403F, binds more SR-B1 than the same amount of 1a154 sE2 in the presence of non-saturating concentrations of HC33.4 (Fig 8D and 8E).

As our ability to query larger sets of naturally occurring HCV isolates for their sensitivity to bNAbs increases, so does our understanding of determinants of bNAb resistance—a key barrier to developing an effective prophylactic vaccine against HCV. In a previous report, we found that bNAbs cluster into functional groups with respect to the HCV variants that they neutralize most and least potently. This clustering of bNAbs is determined at least in part by shared or overlapping binding epitopes, but we and others have shown that polymorphisms distant from known binding epitopes can also confer bNAb resistance [20,22,24]. This study provides evidence that these extra-epitopic polymorphisms play an important role in neutralization resistance of natural E1E2 isolates.

Mutations arising within mAb binding epitopes tend to be an antibody-specific resistance mechanism, and cannot confer resistance to bNAbs with epitopes that are 100% conserved. Here we describe a novel mechanism that can confer resistance to multiple anti-HCV bNAbs, even if the bNAb binding epitopes are completely intact, by modulating E2 binding to SR-B1. To our knowledge, L403F and L438V are the first examples of a naturally-occurring mutations that confer resistance or sensitivity to bNAbs by this mechanism. L438 falls near the CD81 binding site of E2 [40], which is consistent with our finding that mutation at this site reduced sE2 binding to CD81, possibly also contributing to the increased bNAb sensitivity of L438V mutants. Introduction of L438V significantly decreased E1E2 fitness to mediate HCVpp or HCVcc entry into hepatoma cells (S5 Fig), which is consistent with the observed reduction in binding of 1a154_L438V sE2 to CD81 and SR-B1. We found that L403F increased binding to SR-B1 but decreased binding to CD81. Notably, despite these opposing binding effects, we observed a net increase in E1E2 resistance to neutralization by HC33.4 and AR4A after introducing this mutation. The effect of L403F on SR-B1 binding may be dominant over the CD81-binding effect because the interaction of E2 with SR-B1 most likely occurs before binding of CD81 during HCV entry [41,42], or because of differences in relative expression of SR-B1 and CD81 on the surface of hepatocytes. This warrants further study, as it has potentially interesting implications for strategies to inhibit HCV entry with antibodies or small molecules.

The binding epitope of HC33.4 has been mapped in prior studies, and L403 and L438 were not found to be binding residues [28]. The binding epitope of AR4A is less clearly defined, but L403 and L438 were also not among probable AR4A binding residues [26]. Notably, L438 was identified as a contact residue for mAb AR3C in the crystal structure of AR3C/strain H77 E2 described by Kong et al [39], and another study showed that AR3C and AR4A do not compete for binding to E2 [26], Together, these data are all consistent with our finding that L403 and L438 are extra-epitopic for HC33.4 and AR4A.

Recent crystallization of the E2 protein core in complex with a bNAb has been informative [39]. However, large deletions in E2 to facilitate crystallization preclude analysis of many bNAb epitopes, including the HC33.4 and AR4A epitopes. Given the difficulty and limitations of co-crystallizing HCV bNAbs with HCV E2, much of what we know about bNAb-E1E2 interactions will need to be inferred by a comprehensive approach including binding studies with alanine-scanning mutants as well as binding competition assays. This study shows the utility of an additional, complementary approach that can be used to measure neutralizing breadth, group functionally similar bNAbs, and identify bNAb resistance polymorphisms that may fall within or outside of known binding epitopes.

These data are particularly relevant given studies in animal models suggesting that combinations of bNAbs may be necessary to provide sterilizing protection against HCV infection [43,44]. Based on their distinct binding epitopes, it would have been reasonable to assume that neutralizing breadth of bNAbs like HC33.4 and AR4A would be greater if they were used in combination. That may still be true, but this study shows that an unexpectedly high proportion of HCV variants with resistance to one bNAb may also have resistance to the other, which could reduce the efficacy of this bNAb combination.

While we were able to identify polymorphisms modulating resistance to multiple bNAbs, there were limitations to the study design and approach. We only sampled 97% of the naturally occurring polymorphisms that exist at a ≥5% threshold in a large set of Genbank HCV genotype 1 sequences. When the frequency threshold for polymorphism prevalence is reduced to ≥1%, the coverage is reduced to 78%. While SNAPR correctly predicted the 438 locus as a modulator of HC33.4 and AR4A resistance, it incorrectly predicted that L438V would confer bNAb resistance, when in fact this mutation confers increased sensitivity to both bNAbs. The error may have arisen due to genetic linkage between the 438 locus and other resistance-determining loci in E1E2, since the LASSO algorithm, which adjusts for linkage, did not predict that the 438 locus is a determinant of neutralization sensitivity. Notably, the SNAPR algorithm accurately predicted position 408, a known binding residue, as a mediator of HC33.4 resistance, while LASSO did not. Further testing would be necessary to more clearly determine whether SNAPR, LASSO, or a combination of the two methods would be best suited to predict resistance polymorphisms in HCV E1E2.

While the effects of the L403F and L438V polymorphisms are significant, they are small in magnitude relative to the large variation in neutralization sensitivity observed between natural isolates, suggesting that combinations of polymorphisms likely play an important role in bNAb resistance. Even larger, more diverse E1E2 panels are required to reduce confounding from genetic linkage, to probe rarely occurring natural polymorphisms, and to better define the influence of combinations of polymorphisms on neutralization resistance.

In conclusion, we have developed a large, diverse HCV neutralization panel and a statistical approach using amino acid sequence variation and neutralization sensitivity to identify bNAb resistance polymorphisms in E1E2. Despite conservation of HC33.4 and AR4A binding epitopes across the E1E2 panel, we discovered variants with resistance to both bNAbs, identifying polymorphisms conferring resistance to each bNAb individually, as well as polymorphisms outside of either binding epitope that modulate resistance to both bNAbs. We determined that two of these polymorphisms, L403F and L438V, modulate resistance to HC33.4 by increasing or decreasing E2 binding to SR-B1, which is a novel mechanism of bNAb resistance. This study highlights the important contribution of extra-epitopic polymorphisms to bNAb resistance, presenting a potential mechanism by which HCV might persist even in the face of an antibody response targeting multiple conserved epitopes. This diverse viral panel and novel computational pipeline are broadly applicable to future studies to define neutralizing antibody breadth, identify functionally-related bNAbs, and define mechanisms of bNAb resistance.

## Materials and methods

### Source of bNAbs

HC33.4 [29] was a gift of Steven Foung (Stanford University School of Medicine, Stanford, California. AR4A [27] was a gift from Mansun Law (The Scripps Research Institute, La Jolla, California, USA).

### HCV E1E2 expression plasmids

Plasma samples obtained from HCV infected subjects in the BBAASH cohort [15,16,45], Irish Anti-D cohort [46], and Swan Project [47] were used to construct a library of genotype 1 E1E2-expressing lentiviral pseudoparticles using a high-throughput production and screening approach. The E1E2 region was PCR amplified from cDNA reverse transcribed from viral RNA purified from subject plasma and cloned into the expression vector pcDNA3.2/V5/Dest (Invitrogen) using Gateway technology in a one-tube BP/LR reaction, as previously described [19].

### High-throughput HCVpp production and infectivity screening

HCVpp were produced by lipofectamine-mediated transfection of HCV E1E2 and pNL4-3.Luc.R-E- plasmids into HEK293T cells (ATCC) in 96-well plates as previously described [19]. Hep3B cells were exposed to transfected 293T supernatants in order to test for the presence of infectious HCVpp, as previously described [19]. HCVpp were considered infectious in the initial screen if infection of Hep3B cells (ATCC) in a 96 well format resulted in greater than 200,000 RLU of luciferase activity, which is >10X typical values obtained from infection with mock pseudoparticles. E1E2 variants included in the panel differed by at least one amino acid from every other clone contained in the library. Envelopes that displayed enhanced infection in the presence of neutralizing bNAbs (F_u_ >1.2 with either bNAb) were not included in the analysis or in the description of library meta-data as these values most often resulted from HCVpp with poor infectivity. 18 of the 113 E1E2 variants in the final panel were previously described: 1a38, 1a53, 1a72, 1a80, 1a114, 1a123, 1a129, 1a142, 1a154, 1a157, 1b09, 1b14, 1b20, 1b34, 1b38, 1b52 [19] and 1a116, 1b21 [24]. Sequences of the remaining 95 E1E2 clones have been submitted to GenBank accession numbers KY565136—KY565230.

### Phylogenetic analysis

Sanger sequencing of the entire length of the cloned E1E2 region was performed. Amino acid sequences from a nucleic acid MUSCLE alignment [48] were used to build a phylogenetic tree. Initial tree(s) for the heuristic search were obtained automatically by applying Neighbor-Join and BioNJ algorithms to a matrix of pairwise distances estimated using a JTT model, and then selecting the topology with superior log likelihood value [49]. All trees are drawn to scale, with branch lengths measured in the number of substitutions per site, and all positions containing gaps and missing data were eliminated. Evolutionary analyses were conducted in MEGA6 [50]. Sequence logos were generated using VisSPAv1.6 (http://sray.med.som.jhmi.edu/SCRoftware/VisSPA/).

### Site directed mutagenesis

Polymorphisms associated with bNAb resistance or sensitivity were introduced into at least two independent E1E2 clones. Mutants were created using the QuikChange Lightning Multi Site-Directed Mutagenesis Kit (Agilent) and Sanger sequencing was performed to verify that all mutants differed from parent clones at only the desired locus.

### HCVpp infectivity measurements

For infectivity and neutralization testing of the panel of 113 HCVpp, 2,000 Hep3B cells per well were plated in 384-well white flat bottom tissue culture plates. For infectivity and neutralization testing of site-directed mutants 8,000 Hep3B cells per well were plated in flat bottom 96-well tissue culture plates and incubated overnight in a humidified CO_2_ incubator at 37°C. Media was removed from the cells the following day and replaced with 50μL of culture supernatant containing HCVpp (96-well plates) or 25μL of HCVpp supernatant (384 well plates). The plates were placed in a CO_2_ incubator at 37°C for 5 hours, after which the HCVpp were removed and replaced with 100μL of phenol-free Hep3B media (96-well plates) or 50μL of phenol-free Hep3B media (384-well plates) and incubated for 72 hours at 37°C. For 96-well plate infections, media was removed from the cells and 50 μL of 1x Cell Culture Lysis Reagent (Promega) added and left to incubate for >5 minutes then 45μL from each well were then transferred to a white, low-luminescence 96-well plate (Berthold) and read in a Berthold Luminometer (Berthold Technologies Centro LB960). Each sample was tested in duplicate. For 384-well plate infections, cells were lysed directly in the culture plate with 25μL of lysis buffer and luciferase activity measured using a BMG Labtech Fluostar Omega luminometer. A mock pseudoparticle (no envelope) was used as a negative control.

### Measurements of HCVpp neutralization

The same procedure used to measure infectivity was employed, except that HCVpp were incubated with 10μg/mL of bNAb or serial dilutions of bNAb at 37°C for 1 hour prior to addition to the Hep3B target cells. Infections were performed in duplicate with the test antibody and nonspecific human IgG, the negative control. Murine Leukemia Virus (MLV) was used as a control for nonspecific neutralization. Fraction Unaffected (F_u_) was calculated as RLU in the presence of test antibody/RLU in the presence of nonspecific human IgG. Each replicate RLU_mAb_ value was divided by the average of two replicate RLU_IgG_ values. % Neutralization was calculated as (1-F_u_)x100%. To estimate the precision of Fu neutralization measurements, we compared Fu neutralization values of HCVpp measured in independent replicate experiments and observed a highly significant correlation (S6 Fig)

### Subject-adjusted Neutralizing Antibody Prediction of Resistance (SNAPR) algorithm

Amino acid alignments were assembled as described in the phylogenetic analysis. To account for the uneven number of the infectious clones per human donor, neutralization values and corresponding E1E2 sequences were selected at random from each human donor and added to the initial data set until all donors were represented by an equal number of isolates. For each position in the alignment, HCVpp were grouped according to the amino acid encoded at that locus. The amino acid at each position associated with greatest bNAb sensitivity was identified by comparing median F_u_ values of the HCVpp in each amino acid group. F_u_ values for HCVpp in the most sensitive amino acid group were then compared to the F_u_ values of HCVpp with any other amino acid at the same position using a Wilcoxon rank-sum test. The resulting p-value is the SNAPR-value associated with that locus. Analysis was implemented using code developed for R, which is freely available upon request.

### Least Absolute Shrinkage and Selection Operator (LASSO) analysis

The LASSO combines a prediction error term (the least squares error) with a model complexity penalty, which regularizes the model coefficients to perform variable selection and prevent over-fitting [37]. The two replicate fraction unaffected values were square root transformed, and the mean of these used as the outcome variable, which the model aims to explain using the amino acid sequence. The amino acids at each site were our explanatory variables, and these were encoded as indicator variables. Leave-one-out cross validation was used to select the optimal LASSO penalty (with the lowest mean-squared error), which gives the coefficients for each amino acid at each site, which were used as the LASSO results throughout. This was performed in R using the Lasso implementation from the package "glmnet" (https://cran.r-project.org/web/packages/glmnet/).

### HCV E1E2 ELISA

BNAb binding to E1E2 was quantitated using an enzyme-linked immuosorbent assay (ELISA) as previously described [51]. 293T cells were transfected with E1E2 expression constructs. 48 hours post-transfection cell lysates were harvested. Plates were coated with 500ng *Galanthus nivalis* (GNA) lectin (Sigma-Aldrich) and blocked with phosphate-buffered saline containing 0.5% Tween 20, 1% non-fat dry milk, and 1% goat serum. E1E2 cell lysates were added. BNAbs were assayed in duplicate 2.5-fold serial dilutions, starting at 10 μg/ml. Binding was detected using HRP-conjuagated anti-human IgG secondary antibody (BD Pharmingen no. 555788). For binding competitions ELISA, E1E2 protein-coated ELISA wells were pre-incubated with 20 μg/ml of either HC33.4 or AR4A (blocking bNAbs), followed by biotinylated HC33.4 or AR4A at a concentration selected to give 50% of maximal binding (EC_50_), with binding of the biotinylated bNAb detected using streptavidin-horseradish peroxidase. A ratio of binding of each biotinylated bNAb in the presence of blocking bNAb divided by binding in the absence of blocking bNAb was calculated.

### Generation of HCVcc chimeras

HCVcc chimeras were generated as previously described [38,52]. Briefly, after digestion of the HCVcc backbone with AfeI (New England Biolabs), 1a154 (H77 strain), 1a154_L438V, and 1a154_L403F E1E2 genes amplified from library plasmids were inserted in frame using In-Fusion cloning (Clontech). 2μg of plasmid DNA was linearized using XbaI (New England Biolabs) then used for in vitro RNA transcription using the T7 MEGAscript kit (Ambion). RNA clean-up was performed using RNeasy mini kit (Qiagen), quantified using a NanoDrop 1000 spectrophotometer (Thermo Scientific), and stored at –80°C. 10μg of RNA was transfected into 1.8e6 Huh7.5.1 cells (a gift of Charles Rice, The Rockefeller University, New York City, New York, USA) using Nucleofector Kit T (Amaxa) and plated in a 6-cm plate. Transfection supernatants were collected 4–11 days later and stored at -80°C. Supernatants were titered by serial dilution and infection of Huh7.5.1 cells.

### HCVcc neutralization assays

HCVcc neutralization assays were performed in triplicate as described elsewhere [38,52]. Briefly, human hepatoma Huh7.5.1 cells were maintained in DMEM supplemented with 10% fetal bovine serum and nonessential amino acids. 10,000 Huh7.5.1 cells per well were plated in flat bottom 96 well tissue culture plates and incubated overnight at 37°C. The following day, HCVcc were mixed with mAb (3-fold dilutions started at 50μg/mL) then incubated at 37°C for 1 hour. Media was removed from the cells and replaced with 50 μL of HCVcc/antibody mixture. The plates were placed in a CO2 incubator at 37°C overnight, after which the HCVcc were removed and replaced with 100μL of Huh7.5.1 media and incubated for 48 hours at 37°C. Medium was then removed and cells were fixed with 4% formaldehyde then stained for HCV NS5A using primary anti-NS5A antibody 9E10 (a gift of Charles Rice, The Rockefeller University, New York City, New York, USA) at 1:2,000 dilution for 1 hour at room temperature. Cells were washed twice with PBS and stained using secondary antibody Alexa Daylight 488–conjugated goat anti-mouse IgG (Life Technologies) at 1:500 dilution for 1 hour at room temperature. Cells were washed twice in PBS and then stored in 100μl PBS at 4°C. Images were acquired and spot forming units were counted for infection in the presence of mAb (HCVccSFUtest) or PBS alone (HCVccSFUcontrol) using an AID iSpot Reader Spectrum operating AID ELISpot Reader version 7.0. Percent neutralization was calculated as 100% x [1-(HCVccSFUtest /HCVccSFUcontrol)].

### Expression of soluble E2

A truncated, soluble form of the 1a154 (H77) strain E2 ectodomain (sE2) that retains antigenticity and function as previously described [39], encompassing residues 384–645, was cloned into a mammalian expression vector (phCMV3_Ig Kappa_HIS, a gift of Leopold Kong, The Scripps Research Institute, La Jolla, California, USA) from plasmids containing H77 structural proteins. The vector allows expression of E2 protein with a C-terminal His tag as well as an N-terminal murine Ig Kappa leader signal for efficient protein secretion. H77 mutants, L403F and L438V, were created as described above and verified by Sanger sequencing. Each E2 construct was co-transfected with pAdvantage (Promega) into HEK293T cells and incubated for 72 hours at 37°C. Supernatant was collected at 48 and 72 hours, passed through a 0.2μm filter, and concentrated using a regenerated cellulose centrifugal filter with a 10kDa cutoff (Amicon).

### Quantitation of relative sE2 protein concentration

Serial 6.25 fold dilutions of each sE2 supernatant beginning with a 1 to 40 dilution were immobilized onto ELISA wells pre-coated with 500 ng Galanthus nivalis lectin (Sigma-Aldrich) and blocked with PBS containing 0.5% Tween 20, 1% nonfat dry milk, and 1% goat serum. Wells were probed with 0.5 μg of a mouse monoclonal anti-6x His-tag antibody (ab18184, Abcam) and quantified using a HRP-conjugated goat anti-mouse IgG secondary antibody (ab97265, Abcam). The EC50 for each sE2 construct was calculated by nonlinear regression analysis and fold differences in EC50 used to normalize sE2 concentration in subsequent experiments.

### sE2 binding to CHO cells

CHO-CD81 and CHO-SR-B1 binding experiments were carried out as previously described [33]. CHO cells expressing recombinant human CD81 or SR-B1 (a gift from Dr. Matthew Evans, Icahn School of Medicine, Mount Sinai, New York) were detached using PBS supplemented with 4mM EDTA and 10% FBS and washed in PBS containing 1% BSA. Cells (2E+05) were pelleted in a 96-well u-bottom plate and re-suspended in 2 fold serial dilutions of each sE2 construct (H77, L403F, and L438V). Following 30 minutes incubation on ice the cells were washed twice and incubated with 0.5 ug of anti-6x His-tag antibody for another 20 minutes on ice. The cells were then washed again, re-suspended in an Alexa fluor 647-labeled goat anti-mouse IgG secondary antibody, and incubated on ice for 15 minutes. After a final wash, the cells were fixed with 1% paraformaldehyde and analyzed on a LSRII (Becton-Dickinson) using FloJo software (Tree Star). For mAb binding-inhibition experiments, sE2 was normalized for protein concentration, then diluted 1:32 for SR-B1 binding, 1:16 for CD81 binding. sE2 was preincubated with serial dilutions of HC33.4 or nonspecific human IgG, then used to stain CHO cells as above.

## Supporting information

S1 Fig Significant positive correlation between neutralization profiles of HC33.4 and AR4A is observed using subtype 1a-only or subtype 1b-only HCVpp. Each point indicates mean fraction unaffected (F_u_) of a single HCvpp by 10 μg/mL of HC33.4 on the x-axis and AR4A on the y-axis, measured in duplicate. Subtype of HCVpp in each panel is indicated. F_u_ is infection in the presence of 10 μg/mL of bNAb/infection in the presence of nonspecific human IgG. R- and p-values determined by Spearman correlation.
(PDF)

S2 Fig SNAPR analysis of all genotype 1 isolates. SNAPR-values across E1E2 determined using all genotype 1 HCVpp neutraglized by HC33.4 or AR4A. Previously defined HC33.4 and AR4A binding epitopes are indicated (blue and pink), as is hypervariable region 1 (HVR1) (gray), and the portion of E2 crystallized by Kong, et al. ‘C’ indicates the core protein and ‘E1’ and ‘E2’ indicate the E1 and E2 envelope proteins respectively.
(PDF)

S3 Fig Quantitation of relative sE2 protein concentrations in cell culture supernatants containing 1a154, 1a154_L403F, or 1a154_L438V sE2. Serial dilutions of each sE2 protein were added to ELISA wells that had been pre-coated with GNA-lectin. Bound sE2 was quantitated using an antibody specific for the C-terminal sE2 Histidine tag and an HRP-conjugated secondary antibody.
(PDF)

S4 Fig Second independent experiment confirming relative binding of 1a154, 1a154_L403F, and 1a154_L438V sE2 to SR-B1-CHO and CD81-CHO cells. Binding of serial dilutions of 1a154, 1a154_L403F, or 1a154_L438V sE2 to CHO-SR-B1 cells or CHO-CD81 cells. Each point was calculated from 10e4 events. Background binding to wild type CHO cells was subtracted from mean fluorescence intensity (MFI) values. sE2 supernatants were normalized for relative sE2 concentration (shown in S3 Fig) prior to dilution.
(PDF)

S5 Fig Introduction of L438V reduces E1E2 fitness, but L403F does not. **(A)** The indicated mutations were introduced into 1a154 (H77) E1E2, and HCVpp with a luciferase reporter gene were produced. These HCVpp were used to infect Hep3B hepatoma cells, and entry quantitated after 72 hours by measurement of relative light units (RLU). Each data point indicates an independent experiment (independent transfection and infection) performed in duplicate. **(B)** Infectivity of HCVcc chimeras expressing 1a154, 1a154_L403F, or 1a154_L438V E1E2, with supernatants harvested Day 4 or Day 11 post RNA transfection. Supernatants were added to Huh7.5.1 hepatoma cells, with infectivity measured by Spot Forming Units (SFU) 48 hours later. Each point represents infectivity of HCVcc from an independent transfection. Groups were compared by one-way ANOVA with correction for multiple comparisons. (ns, not significant; *, p <.0.05, **, p<0.005, ***, p<0.005).
(PDF)

S6 Fig Analysis of experimental variation between Fu measurements. (**A**) Variation between technical replicate measurements of Fu in the presence of nonspecific human IgG for 113 HCVpp in the neutralization screen. Each point represents the mean of duplicate Fu measurements for one HCVpp on the x-axis and the standard deviation between those values on the y-axis. Technical replicate measurements were obtained from two Hep3B wells infected in the same experiment. (**B-C**) Variation between Fu measurements of the same HCVpp/mAb combinations in independent experiments. Each independent experiment was carried out with an independently produced preparation (transfection) of HCVpp and an independent neutralization assay, performed on a different day. (**B**) Correlation between independent experiments. (**C**) Fold difference between independent experiments.
(PDF)

S1 Data Values for infectivity (in relative light units, RLU) of each HCVpp in the panel in the presence of nonspecific human IgG, HC33.4, or AR4A, measured in duplicate. Fu values calculated from these RLU values are also shown, as are the sequence of each E1E2 variant. Each variant in the panel is assigned an arbitrary number to indicate variants that were isolated from the same study subject.
(XLSX)
