# Supplementary figures and images for: Extra-epitopic hepatitis C virus polymorphisms confer resistance to broadly neutralizing antibodies by modulating binding to scavenger receptor B1
Source: PLoS Pathog. 2017 Feb 24;13(2):e1006235. doi: 10.1371/journal.ppat.1006235 (PMC5342271; doi:10.1371/journal.ppat.1006235)

Subtype 1a

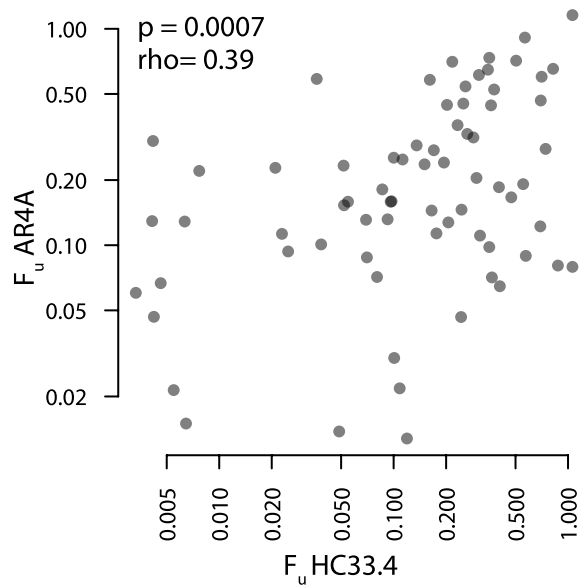

Subtype 1b

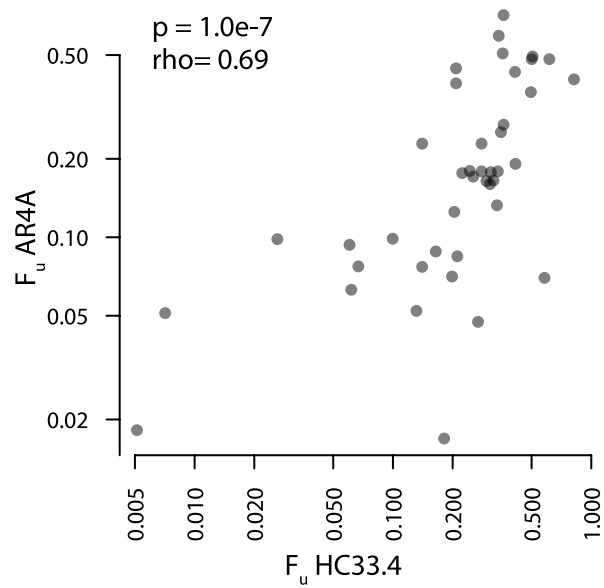

Supplement: S1 Fig — Each point indicates mean fraction unaffected (Fu) of a single HCvpp by 10 μg/mL of HC33.4 on the x-axis and AR4A on the y-axis, measured in duplicate. Subtype of HCVpp in each panel is indicated. Fu is infection in the presence of 10 μg/mL of bNAb/infection in the presence of nonspecific human IgG. R- and p-values determined by Spearman correlation. (PDF) [file ppat.1006235.s001.pdf]

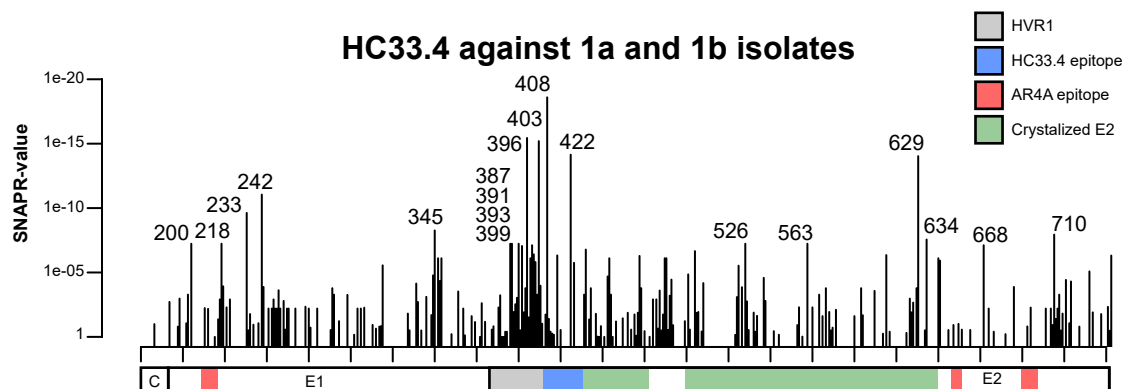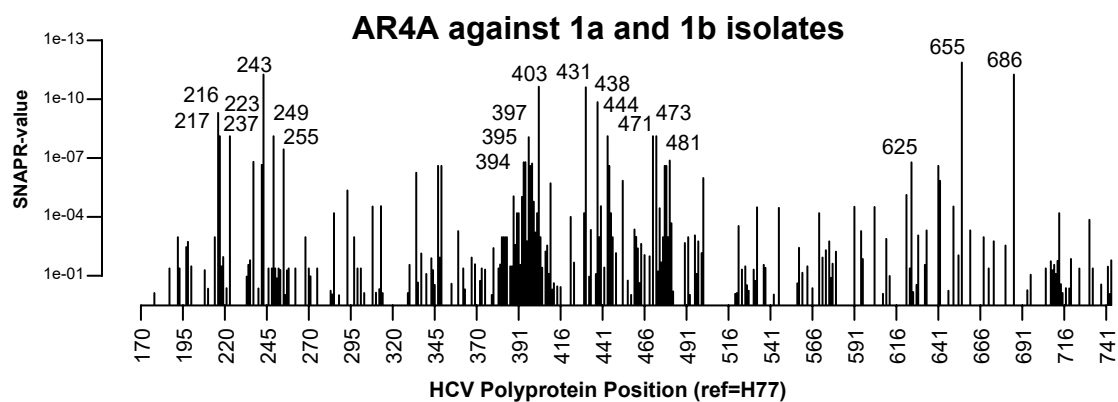

Supplement: S2 Fig — SNAPR-values across E1E2 determined using all genotype 1 HCVpp neutraglized by HC33.4 or AR4A. Previously defined HC33.4 and AR4A binding epitopes are indicated (blue and pink), as is hypervariable region 1 (HVR1) (gray), and the portion of E2 crystallized by Kong, et al. ‘C’ indicates the core protein and ‘E1’ and ‘E2’ indicate the E1 and E2 envelope proteins respectively. (PDF) [file ppat.1006235.s002.pdf]

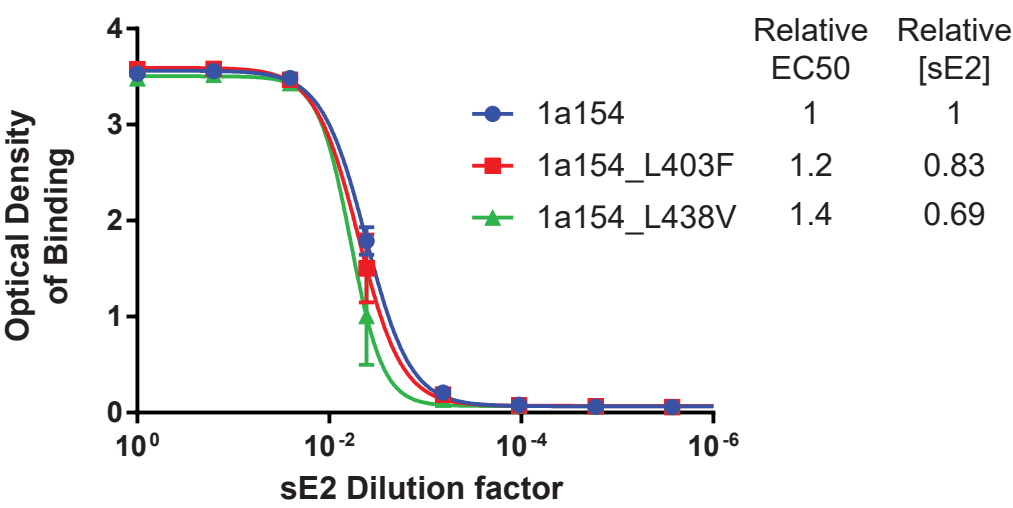

Supplement: S3 Fig — Serial dilutions of each sE2 protein were added to ELISA wells that had been pre-coated with GNA-lectin. Bound sE2 was quantitated using an antibody specific for the C-terminal sE2 Histidine tag and an HRP-conjugated secondary antibody. (PDF) [file ppat.1006235.s003.pdf]

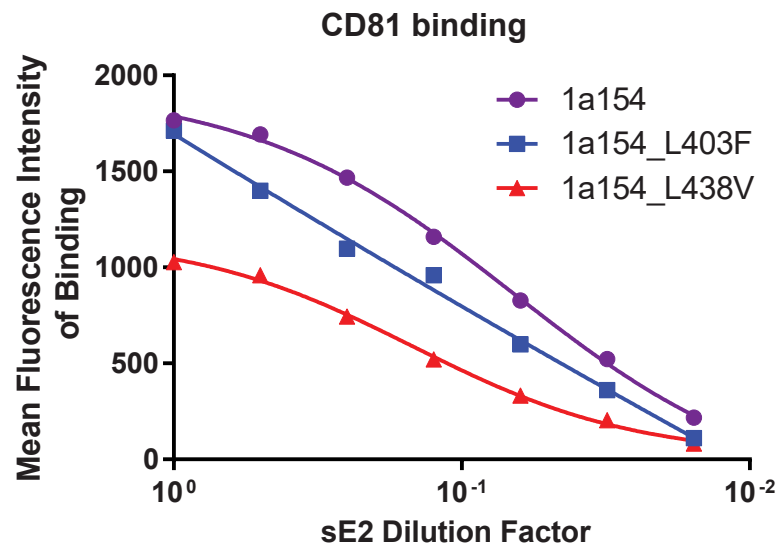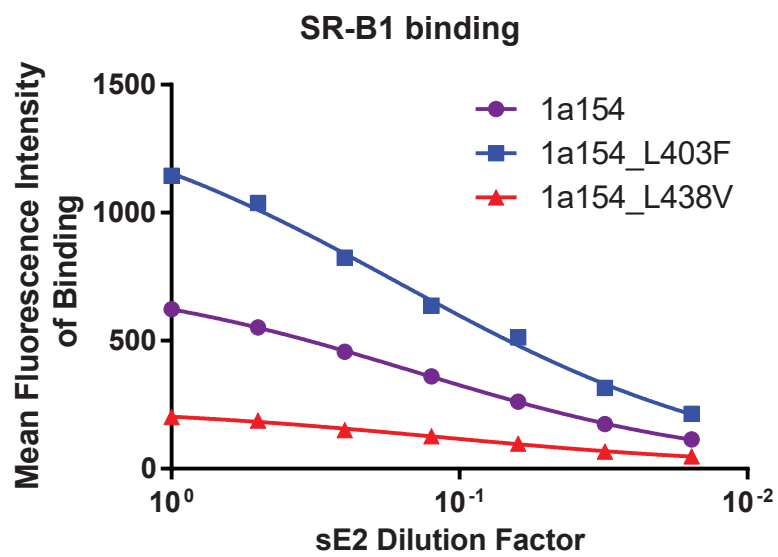

Supplement: S4 Fig — Binding of serial dilutions of 1a154, 1a154_L403F, or 1a154_L438V sE2 to CHO-SR-B1 cells or CHO-CD81 cells. Each point was calculated from 10e4 events. Background binding to wild type CHO cells was subtracted from mean fluorescence intensity (MFI) values. sE2 supernatants were normalized for relative sE2 concentration (shown in S3 Fig) prior to dilution. (PDF) [file ppat.1006235.s004.pdf]

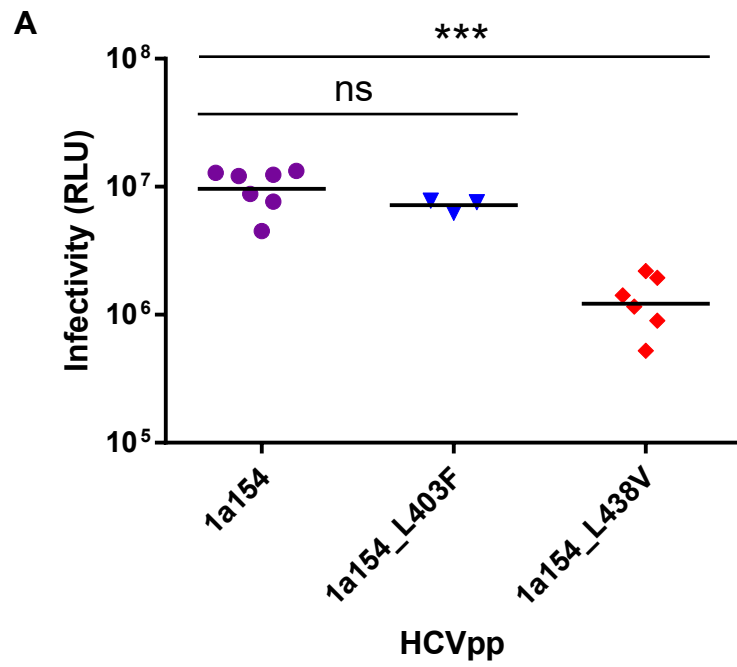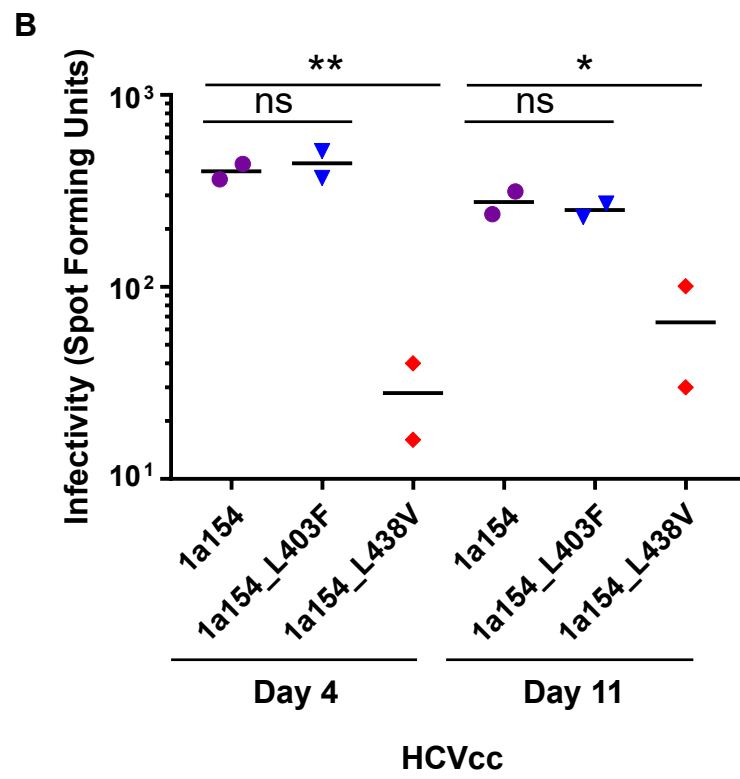

Supplement: S5 Fig — (A) The indicated mutations were introduced into 1a154 (H77) E1E2, and HCVpp with a luciferase reporter gene were produced. These HCVpp were used to infect Hep3B hepatoma cells, and entry quantitated after 72 hours by measurement of relative light units (RLU). Each data point indicates an independent experiment (independent transfection and infection) performed in duplicate. (B) Infectivity of HCVcc chimeras expressing 1a154, 1a154_L403F, or 1a154_L438V E1E2, with supernatants harvested Day 4 or Day 11 post RNA transfection. Supernatants were added to Huh7.5.1 hepatoma cells, with infectivity measured by Spot Forming Units (SFU) 48 hours later. Each point represents infectivity of HCVcc from an independent transfection. Groups were compared by one-way ANOVA with correction for multiple comparisons. (ns, not significant; *, p <.0.05, **, p<0.005, ***, p<0.005). (PDF) [file ppat.1006235.s005.pdf]

**A**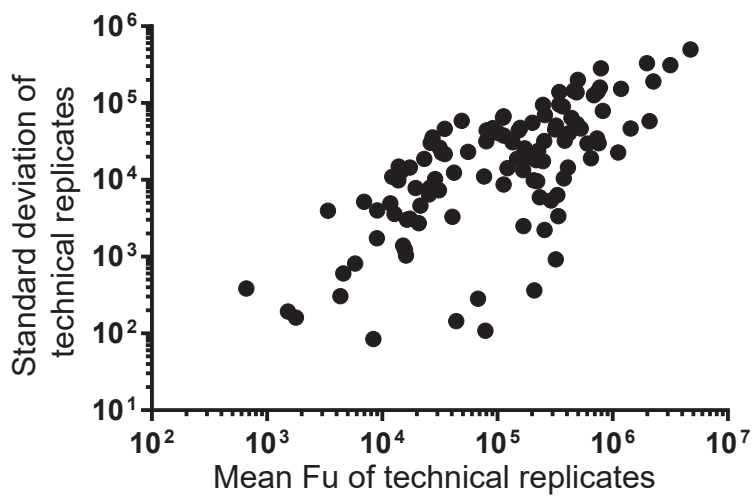**B**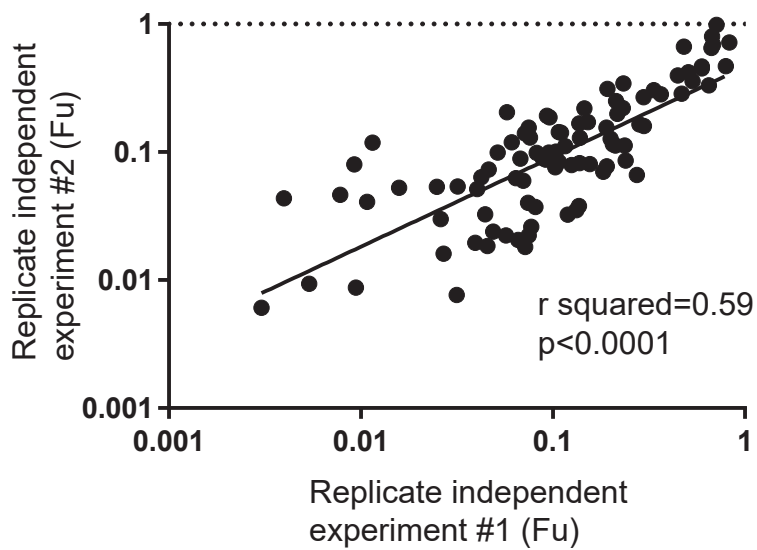**C**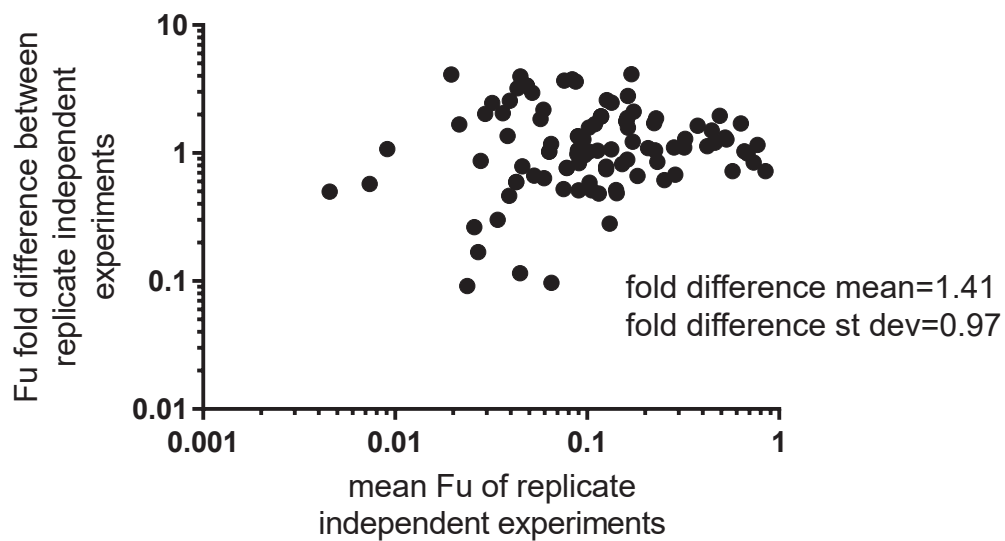

Supplement: S6 Fig — (A) Variation between technical replicate measurements of Fu in the presence of nonspecific human IgG for 113 HCVpp in the neutralization screen. Each point represents the mean of duplicate Fu measurements for one HCVpp on the x-axis and the standard deviation between those values on the y-axis. Technical replicate measurements were obtained from two Hep3B wells infected in the same experiment. (B-C) Variation between Fu measurements of the same HCVpp/mAb combinations in independent experiments. Each independent experiment was carried out with an independently produced preparation (transfection) of HCVpp and an independent neutralization assay, performed on a different day. (B) Correlation between independent experiments. (C) Fold difference between independent experiments. (PDF) [file ppat.1006235.s006.pdf]
